# Core outcome sets are valuable, but methodological evidence can improve robustness

**DOI:** 10.1111/1471-0528.16419

**Published:** 2020-09-29

**Authors:** IM Beune, W Ganzevoort, SJ Gordijn

**Affiliations:** ^1^ Department of Obstetrics and Gynaecology University Medical Center Groningen University of Groningen Groningen The Netherlands; ^2^ Department of Obstetrics and Gynaecology Amsterdam University Medical Centres University of Amsterdam Amsterdam The Netherlands

A core outcome set (COS) is the agreed minimum set of outcomes to be measured in studies regarding a specific topic. A COS is considered to encompass the most relevant outcomes and does not restrict researchers. One should realise that outcomes not included in the COS may actually be important for specific research questions and different study designs.

The COMET handbook (Core Outcome Measures in Effectiveness Trials) (Williamson et al. *Trials* 2017;18[Suppl 3]:280), used in the current study (Duffy et al *BJOG* 2020; https://doi.org/10.1111/1471‐0528.16319), describes consensus methodology for COS development. In a nutshell, it is advised to start with a systematic review to identify all possible outcomes; then use the Delphi strategy to converge opinions to consensus; and finally, the prioritised list of outcomes is discussed in a face‐to‐face consensus meeting in which the final COS is conducted. The team of Duffy et al. (*BJOG* 2020; 127:1516–26) have developed an important COS using this methodology, meeting all quality recommendations for COSs as formulated in COS‐STAD, and we commend them for it (Kirkham et al. *PLoS Med* 2017;14[11]:e1002447).

We would like to raise the point that some elements of the COMET methodology for COS development are by agreement rather than proven methodology and we suggest that alternatives may be considered.

1. It remains unknown whether a systematic review is preferable over a scoping review. A scoping review is advised to clarify key concepts in the literature (Munn et al. *BMC Med Res Methodol* 2018;18:143); chances are low that an outcome that requires a systematic review to identify it, is fundamental for all research in the field.
2. COMET states that a response rate of 80% for each stakeholder group is typical, but there is no frame of reference to establish what attrition rate is acceptable to avoid losing the strength of the panel. Did the drop‐out of 37% of the total group in the Delphi rounds in this study have a significant effect on the final COS?
3. The crucial contribution of lay experts is recognised by COMET but there is no advice as to the number or percentage of lay experts in a panel. In previous COS procedures, the contribution of lay experts varied from 4 to 50% (Williamson et al. *Trials* 2017;18[Suppl 3]:280).
4. A consensus meeting facilitates acceleration of the consensus building procedure because the panel members are in direct contact and clarifications are readily available. However:
  - In contrast to the online Delphi procedure, a ‘strong voice’ may affect voting behaviour, particularly when patients or lay experts are impressed with knowledgeable professional experts.
  - In this study, 47 outcomes were presented to participants in the consensus meeting; ultimately 22 outcomes (including four newly introduced outcomes) were selected. It is unknown whether an electronic meeting (international and COVID‐19 proof) may reduce such selection bias.
  - A consensus‐meeting at the end of a Delphi procedure may have a major impact, as it is not known whether the original panel agrees with the final COS. A consensus meeting held at the beginning or between Delphi rounds may have a different impact.

Delphi and COMET methodologies are valid and valuable tools for consensus building, particularly because a COS is never (only) a gold standard. As there is also no gold standard of the methodology, it remains pivotal to appreciate the strengths and vulnerabilities of the methodology by doing studies that strengthen the COMET and Delphi methodologies.

## Disclosure of interests

All authors have no conflict of interests to declare. Completed disclosure of interest forms are available to view online as supporting information.

## Supporting information

Supplementary Material

Supplementary Material

Supplementary Material

